# Aspect of New Legislation

**Published:** 1895-06

**Authors:** 


One Aspect of New Legislation.—This has been a fruitful year for legislation, and will pave the way for more stringent laws in the very near future, as well as point the way in other States. The success attained in New York and Pennsylvania, preceded by the statutes of Maryland, California, Ohio, and New Jersey, with prospective legislation in the same direction in Massachusetts, Maine, Wisconsin, Illinois, Missouri, Virginia, and West Virginia, all of which shall tend to throw stronger safeguards around the future practitioners of these several States, and be a surer means of protecting the public from the continual dangers of empiricism and quackery. These changes mean much to the institutions of veterinary learning, and to those who still cling to the short course restricts very much the field for recruiting their new classes. Canada has also joined in the agitation, and the meeting at Listowel, Ontario, this past winter, was only the first ebullition of the slumbering fires of agitation among those who will appreciate the fact that the courses of veterinary instruction in all schools and colleges must be broader, deeper, and better. It will well bear repetition, that we no longer have need of an increase of the present imperfectly-educated veterinarian, whose sphere might be said to be limited to the treatment of the horse. I speak for a large number of the profession in practice to-day, when I say that none more fully realize their own deficiencies in the stronger men demanded than these practitioners themselves. With the great field of sanitary work and investigation, the food supply in its many aspects, the disposition to accept no longer the present conclusions relative to certain diseases in the domesticated animals, which annually cause great losses, and which must soon be solved in the interests of those on whom these great losses fall. These are only a few of the thoughts which suggest themselves in view of this legislation, and will afford a fruitful theme for consideration at the coming meeting of the Association of Faculties of North America.

